# U3 snoRNA‐mediated degradation of ZBTB7A regulates aerobic glycolysis in isocitrate dehydrogenase 1 wild‐type glioblastoma cells

**DOI:** 10.1111/cns.14218

**Published:** 2023-04-17

**Authors:** Weiwei Dong, Yunhui Liu, Ping Wang, Xuelei Ruan, Libo Liu, Yixue Xue, Teng Ma, Tiange E, Di Wang, Chunqing Yang, Hongda Lin, Jian Song, Xiaobai Liu

**Affiliations:** ^1^ Department of Neurosurgery Shengjing Hospital of China Medical University Shenyang China; ^2^ Key Laboratory of Neuro‐oncology in Liaoning Province Shenyang China; ^3^ Liaoning Medical Surgery and Rehabilitation Robot Technology Engineering Research Center Shenyang China; ^4^ Department of Neurobiology, School of Life Sciences China Medical University Shenyang China

**Keywords:** aerobic glycolysis, glioblastoma, IDH1, U3, ZBTB7A

## Abstract

**Aims:**

The isocitrate dehydrogenase (IDH) phenotype is associated with reprogrammed energy metabolism in glioblastoma (GBM) cells. Small nucleolar RNAs (snoRNAs) are known to exert an important regulatory role in the energy metabolism of tumor cells. The purpose of this study was to investigate the role of C/D box snoRNA U3 and transcription factor zinc finger and BTB domain‐containing 7A (ZBTB7A) in the regulation of aerobic glycolysis and the proliferative capacity of IDH1 wild‐type (IDH1^WT^) GBM cells.

**Methods:**

Quantitative reverse transcription PCR and western blot assays were utilized to detect snoRNA U3 and ZBTB7A expression. U3 promoter methylation status was analyzed via bisulfite sequencing and methylation‐specific PCR. Seahorse XF glycolysis stress assays, lactate production and glucose consumption measurement assays, and cell viability assays were utilized to detect glycolysis and proliferation of IDH1^WT^ GBM cells.

**Results:**

We found that hypomethylation of the CpG island in the promoter region of U3 led to the upregulation of U3 expression in IDH1^WT^ GBM cells, and the knockdown of U3 suppressed aerobic glycolysis and the proliferation ability of IDH1^WT^ GBM cells. We found that small nucleolar‐derived RNA (sdRNA) U3‐miR, a small fragment produced by U3, was able to bind to the ZBTB4 3′UTR region and reduce ZBTB7A mRNA stability, thereby downregulating ZBTB7A protein expression. Furthermore, ZBTB7A transcriptionally inhibited the expression of hexokinase 2 (HK2) and lactate dehydrogenase A (LDHA), which are key enzymes of aerobic glycolysis, by directly binding to the HK2 and LDHA promoter regions, thereby forming the U3/ZBTB7A/HK2 LDHA pathway that regulates aerobic glycolysis and proliferation of IDH1^WT^ GBM cells.

**Conclusion:**

U3 enhances aerobic glycolysis and proliferation in IDH1^WT^ GBM cells via the U3/ZBTB7A/HK2 LDHA axis.

## Graphical abstract

The aerobic glycolysis and proliferation of IDH1^WT^ GBM cells are significantly higher than those of IDH1^R132H^ GBM cells. U3/ZBTB7A/HK2 LDHA pathway regulates aerobic glycolysis and proliferation of IDH1^WT^ GBM cells. U3 snoRNA may be a molecular target for the treatment of IDH1^WT^GBM.

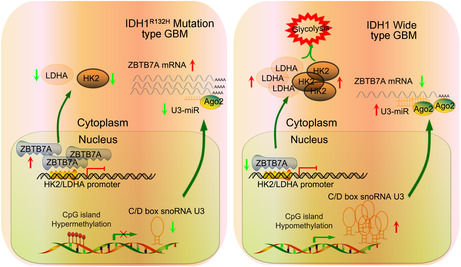

## 1 INTRODUCTION

At present, glioblastoma (GBM) is the most malignant intracranial tumor, with high morbidity and mortality rates.^1^, ^2^, ^3^ The traditional World Health Organization (WHO) classification of gliomas does not accurately reflect the heterogeneity of GBM and thus, cannot be used to accurately guide treatment options or assess patient prognosis. The 2016 updated WHO classification of central nervous system tumors added molecular phenotypes to the traditional histological classification. It has been reported that the molecular phenotypes of GBM are closely related to disease progression and the prognosis of GBM patients. The current predominant molecular phenotype classification of GBM is based on single amino acid missense mutations in isocitrate dehydrogenase1 (IDH1), which facilitates the classification of GBM into IDH1 wild‐type (IDH1^WT^) and IDH1 mutant‐type (IDH1^Mut^). IDH1 R132H (IDH1^R132H^) is the most common point mutation.^4^, ^5^, ^6^ Active aerobic glycolysis, known as the Warburg effect, is a typical metabolic feature of GBM cells. Glycolysis can provide energy and raw materials for the rapid proliferation of GBM cells.^7^, ^8^ Studies have found that the aerobic glycolytic and proliferative capacity of IDH1^WT^ GBM cells are significantly higher than that of IDH1^Mut^ GBM cells, and IDH1^WT^ GBM has been associated with a shortened survival time compared with IDH1^Mut^ GBM.^6^, ^9^ Therefore, it is essential that the molecular mechanisms regulating aerobic glycolysis in IDH1^WT^ GBM cells are studied as they may reveal novel mechanisms of malignant tumorigenesis of IDH1^WT^ GBM from the perspective of glycolytic reprogramming.

IDH1 catalyzes the generation of ɑ‐ketoglutarate (ɑ‐KG) from isocitric acid, whereas IDH1 mutations result in abnormal enzyme activity that converts ɑ‐KG to D‐2‐hydroxyglutarate (D‐2‐HG). D‐2‐HG accumulation suppresses ɑ‐KG‐dependent DNA demethylase activity. IDH1 mutations are closely correlated with the glioma‐CpG island methylator phenotype (G‐CIMP),^4^, ^10^ whereas IDH1^WT^ GBM is commonly associated with the hypomethylation of CpG islands. It has been reported that the methylation status of CpG islands in DNA promoter regions is closely linked with the expression of small nucleolar RNA (snoRNA).^11^ snoRNAs, which are noncoding small RNAs, are located mainly in the nucleus and participate in the tumorigenesis of various tumors.^12^, ^13^ For example, SNORD44 is generally expressed at low levels in gliomas; however, the high expression of SNORD44 inhibits GBM proliferation, invasion, and migration and promotes apoptosis.^14^ SNORD12B expression is elevated in GBM and promotes glycolipid metabolism and proliferation.^15^ U3 belongs to the RNU3 nuclear small RNA family, which is located at the core of the nonhomologous recombinant palindrome sequence on the long arm of chromosome 17 and is closely correlated with tumorigenesis.^16^, ^17^ Currently, the differential expression of U3 in IDH1^WT^ and IDH1^Mut^ GBM and the mechanisms regulating aerobic glycolysis in IDH1^WT^ GBM remain unclear.

The transcription factor ZBTB7A (zinc finger and BTB domain‐containing 7A) is a member of the POK (POZ/BTB and Krüppel) protein family and is known to repress target genes by recruiting co‐repressors.^18^ In osteosarcoma, ZBTB7A represses linc00473 transcription and expression and regulates the sensitivity of osteosarcoma cells to cisplatin chemotherapy.^19^ In melanocytes, ZBTB7A transcriptionally represses the expression of the key adhesion protein MCAM and regulates the migratory and invasive abilities of melanoma cells.^20^ However, there have been no reports on the role of ZBTB7A in aerobic glycolysis and the proliferative capacity of IDH1^WT^ GBM cells. Hexokinase 2 (HK2) and lactate dehydrogenase A (LDHA) are key enzymes that regulate aerobic glycolytic energy metabolism. Increased expression of HK2 and LDHA in GBM cells can promote the aerobic glycolytic capacity of cells, which in turn can promote cell proliferation, migration, and invasion.^7^, ^8^, ^21^

In the present study, we demonstrated the differential expression of U3 and ZBTB7A in IDH1^WT^ and IDH1^Mut^ GBM tissues and cells, as well as the intermolecular interactions between them. Furthermore, we explored the mechanism responsible for their effects on aerobic glycolysis and the proliferative capacity of IDH1^WT^ GBM cells. This study identified a new mechanism of tumorigenesis and explored new molecular targets for the treatment of IDH1^WT^ GBM from the perspective of aerobic glycolysis.

## 2 MATERIALS AND METHODS

### 2.1 Clinical specimens

The normal brain specimens and glioma specimens used in this study were collected from the Department of Neurosurgery, Shengjing Hospital Affiliated China Medical University. This study received ethical approval by the medical ethics committee of the Shengjing Hospital Affiliated China Medical University. For more detailed information, refer to Supporting Information of Materials and Methods.

### 2.2 Cell lines

Human embryonic kidney (HEK) 293 T cells and human GBM cell lines (U87 and U251) were purchased from Institutes for Biological Sciences Cell Resource Center (Shanghai, China). Normal human astrocyte (NHA) cells were purchased from the ScienCell Research Laboratories (CA, USA). For more detailed information, refer to Supporting Information of Materials and Methods.

### 2.3 Quantitative real‐time PCR (qRT‐PCR)

RNA was extracted from tissues and cells via Trizol reagent (Life Technologies Corporation, Carlsbad, CA, USA) according to the manufacturer's protocol. RNA expression was measured using the method of qRT‐PCR. The primers are presented in Table S1. For more detailed information, refer to Supporting Information of Materials and Methods.

### 2.4 Western blot

Western blot was performed as previously described.^22^, ^23^ For more detailed information about experimental procedure and antibodies information, please refer to Supporting Information of Materials and Methods.

### 2.5 Cell transfection

U87^R132H^ and U251^R132H^ cells were obtained from transduction of plasmid carrying IDH1 R132H variant into U87 and U251 cells.^24^, ^25^ The short‐hairpin RNA directed against ZBTB7A(ZBTB7A(−)) and nontargeting short‐hairpin RNA(ZBTB7A(−)NC), the short‐hairpin RNA directed against Dicer(Dicer(−)) and nontargeting short‐hairpin RNA(Dicer(−)NC), ZBTB7A full length(ZBTB7A(+)) plasmid and its negative control plasmid(ZBTB7A(+)NC) were synthesized by Gene‐Pharama (Shanghai, China). The sequences for the short‐hairpin RNA targeting sequence of ZBTB7A and Dicer were presented in Table S2. U3 sgRNA CRISPR/Cas9 All‐in‐One plasmid (U3(−)) and its nontargeting sgRNA (U3(−)NC), U3 full length(U3(+)) plasmid and its negative control plasmid(U3(+)NC) were synthesized by Syngen Tech (Beijing, China). The sgRNA sequences were presented in Table S3. The qRT‐PCR and western blot were utilized to confirm transfection efficacy (Figure S1). For more detailed information about experimental procedure, please refer to Supporting Information of Materials and Methods.

### 2.6 Extracellular acidification rate

Extracellular acidification rate (ECAR) assay was performed as previously described.^22^, ^23^ For more detailed information about experimental procedure, please refer to Supporting Information of Materials and Methods.

### 2.7 Glucose utilization and lactate production assays

Glucose utilization and lactate production was measured as previously described.^22^, ^23^ For more detailed information about experimental procedure, please refer to Supporting Information of Materials and Methods.

### 2.8 CCK‐8

Cell proliferation was assessed via CCK‐8 assay as previously described.^22^, ^23^ For more detailed information about experimental procedure, please refer to Supporting Information of Materials and Methods.

### 2.9 Colony formation assay

Following digestion, 1 × 10^3^ cells per well were seeded in 6 cm dishes. The cells were fixed with methanol and stained with crystal violet solution after 3 weeks of culture. After staining for 0.5 h, the colonies were examined and counted.

### 2.10 U3 promoter methylation analysis

DNA was extracted using the QIAamp DNA Mini Kit (Qiagen, Hilden, Germany) and bisulfite converted using the EpiTect Bisulfite Kit (Qiagen, Hilden, Germany) according to the manufacturer's protocol. Bisulfite‐transformed DNA was used for methylation‐specific PCR (MSP) to identify U3 promoter methylation status. Amplified PCR products were separated by 3% agarose gel electrophoresis and visualized by UVP Biospectrum imaging system. Bisulfite sequencing PCR (BSP) analysis of the U3 promoter gene was accomplished by Sangon Biotech Co., Ltd. (Shanghai, China). 5‐aza‐2′‐deoxycytidine (Sigma‐Aldrich, StLouis, MO, USA), a DNA methylation inhibitor, was used at a concentration of 10 μM to block DNA methylation. The primer for the MSP was designed on free‐on‐line MethPrimer website (http://www.urogene.org/methprimer/) and listed in Table S4.

### 2.11 Northern blot

Northern blot procedure was performed with the Signosis High Sensitive miRNA Northern Blot Assay Kit (Signosis Inc., Santa Clara, CA, USA) following the manufacturer's protocol. Total RNA (20 μg) was separated on 10% acrylamide denaturing gel and then blotted onto a nylon Hybond N membrane and analyzed using oligonucleotides probe complementary to U3‐miR.

### 2.12 RNA immunoprecipitation assay

The RNA immunoprecipitation (RIP) assay was performed as previously described.^22^, ^23^ For more detailed information about experimental procedure, please refer to Supporting Information of Materials and Methods.

### 2.13 RNA stability measurement

RNA de novo synthesis was blocked by actinomycin D (Sigma, St Louis, MO, USA). Total RNA was isolated at various time points and quantified using qRT‐PCR. The half‐life of RNA is measured by its level decreasing to 50% at a specific time point when compared to zero time.

### 2.14 Chromatin immunoprecipitation assay

Chromatin immunoprecipitation (ChIP) assay was performed via Simple ChIP Enzymatic Chromatin IP Kit (Cell Signaling Technology, Danvers, MA, USA) as described previously.^22^, ^23^ For more detailed information about experimental procedure, please refer to Supporting Information of Materials and Methods. The primers used for ChIP assay are listed in Table S5.

### 2.15 Dual‐luciferase reporter assay

Dual‐luciferase reporter assay was performed as previously described.^22^, ^23^ For more detailed information about experimental procedure, please refer to Supporting Information of Materials and Methods.

### 2.16 Tumor xenograft in nude mouse

Animal experiments were performed as previously described.^22^, ^23^ The stable transfected and expressing cells were utilized to establish xenograft models in nude mice. For more detailed information about experimental procedure, please refer to Supporting Information of Materials and Methods.

### 2.17 Statistical analysis

All values are presented as mean ± standard deviation (SD). Each experiment was conducted three times independently. The normality of the data was analyzed using the Shapiro–Wilk test. The data (two groups) were analyzed using the Student's *t*‐test. The data (more than two groups) were analyzed using one‐way or two‐way ANOVA analysis of variance followed by the Dunnett's multiple comparisons test or Sidak's multiple comparisons test. For data not normally distributed, nonparametric tests were used. Statistical analysis was conducted via GraphPad Prism v8.4, and *p* value <0.05 was considered statistically significant.

## 3 RESULTS

### 3.1 The glycolytic capacity and proliferation of IDH1^WT^ GBM cells were significantly higher than those of IDH1^R132H^ GBM cells

**FIGURE 1:**
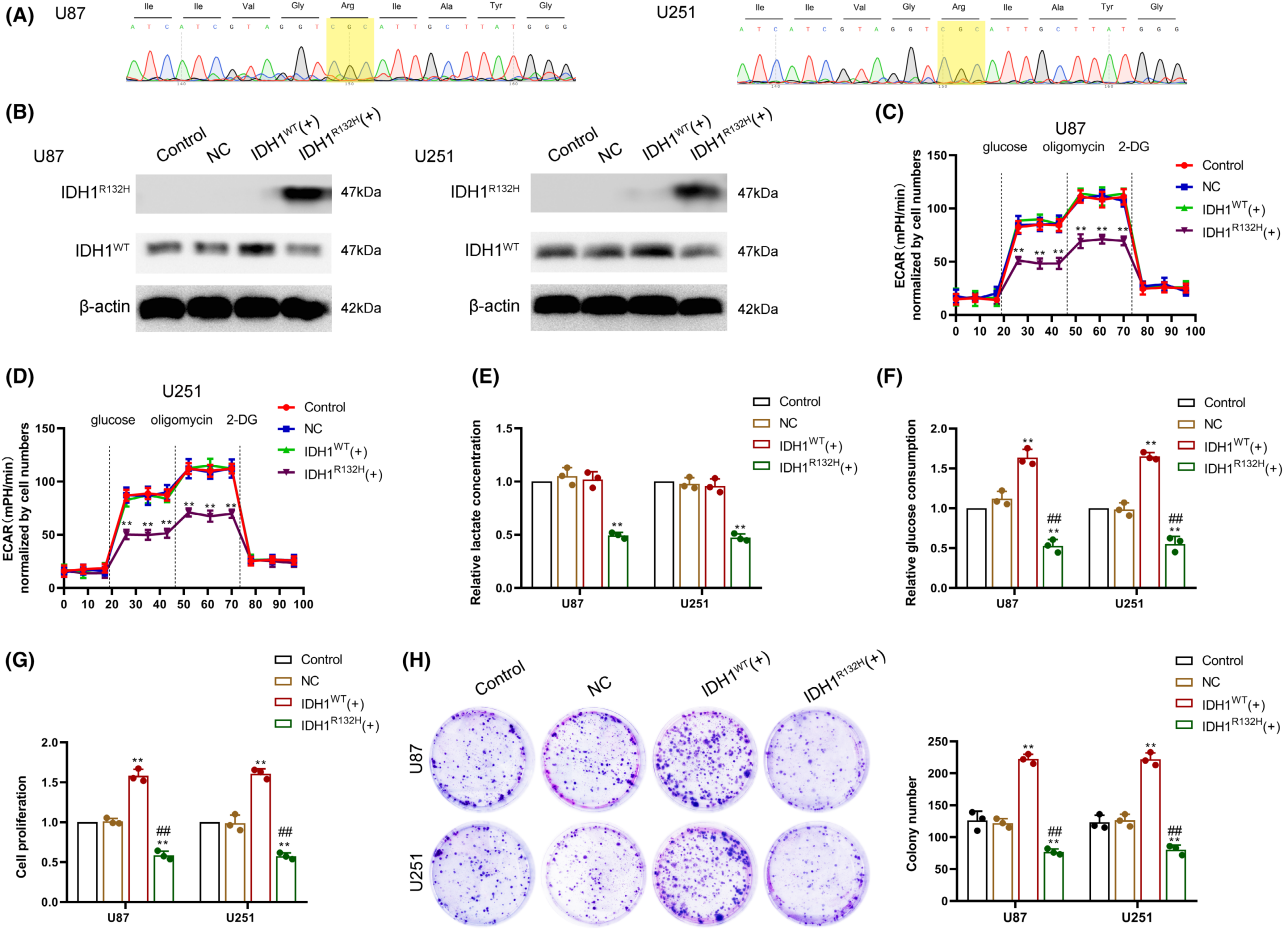
The isocitrate dehydrogenase 1 wild‐type (IDH1^WT^) molecular phenotype is correlated with enhanced aerobic glycolysis and proliferation ability. (A) U87 and U251 glioblastoma (GBM) cell lines were identified as IDH1^WT^ via Sanger sequencing for the IDH1 codon 132. (B) Western blot analysis detected the indicated proteins in control, empty vector (negative control, NC), IDH1^WT^ vector, and IDH1^R132H^ vector transfected U87 and U251 cells using IDH1^R132H^ antibodies and IDH1^WT^ antibodies. (C) Aerobic glycolytic ability was measured using extracellular acidification rate (ECAR) assay in the abovementioned transfected U87 cell. (D) Aerobic glycolytic ability was measured using ECAR assay in the abovementioned transfected U251 cell. (E) Lactate production was measured in the abovementioned transfected cells. (F) Glucose consumption was measured in the abovementioned transfected cells. (G) Cell viability was analyzed in the abovementioned transfected cells using CCK‐8 assay. (H) The proliferation ability of the abovementioned transfected cells was analyzed using a colony formation assay. **p < 0.01 versus NC group; **p < 0.01 versus IDH1^WT^(+) group. Data are presented as the mean ± SD of three independent experiments per group, unless otherwise specified. Data were statistically analyzed using one‐way analysis of variance (ANOVA).

Sanger sequencing was used to confirm that the GBM cell lines, U87 and U251 were IDH1^WT^ GBM cells (Figure 1A). IDH1^R132H^ and IDH1^WT^ overexpression vectors were stably transfected into GBM cells, and western blot experiment detected the indicated proteins IDH1^R132H^ and IDH1^WT^ expression using IDH1^R132H^ and IDH1^WT^ antibodies (Figure 1B). Subsequently, we analyzed the differences in aerobic glycolytic and proliferative capacity among control, empty vector (negative control, NC), IDH1^WT^ vector, and IDH1^R132H^ vector transfected U87 and U251 cells. The aerobic glycolytic capacity of IDH1^WT^ GBM cells was significantly higher than that of IDH1^R132H^ GBM cells, while overexpression of IDH1^WT^ did not increase aerobic glycolytic capacity in IDH1^WT^ GBM cells (Figure 1C–E). Glucose consumption was significantly higher in IDH1^WT^ GBM cells than in IDH1^R132H^ GBM cells, and the overexpression of IDH1^WT^ increased glucose consumption (Figure 1F). Similarly, IDH1^WT^ GBM cells had a significantly higher proliferative capacity than IDH1^R132H^ GBM cells, and the overexpression of IDH1^WT^ enhanced cell proliferation capacity (Figure 1G,H). The above results demonstrated that IDH1^WT^ GBM cells had higher aerobic glycolytic and proliferative capacity when compared to IDH1^R132H^ GBM cells. Overexpression of IDH1^WT^ enhanced cell proliferation but did not increase aerobic glycolytic capacity.

### 3.2 U3 was highly expressed in IDH1^WT^ GBM and U3 knockdown inhibited glycolytic capacity and proliferation

**FIGURE 2:**
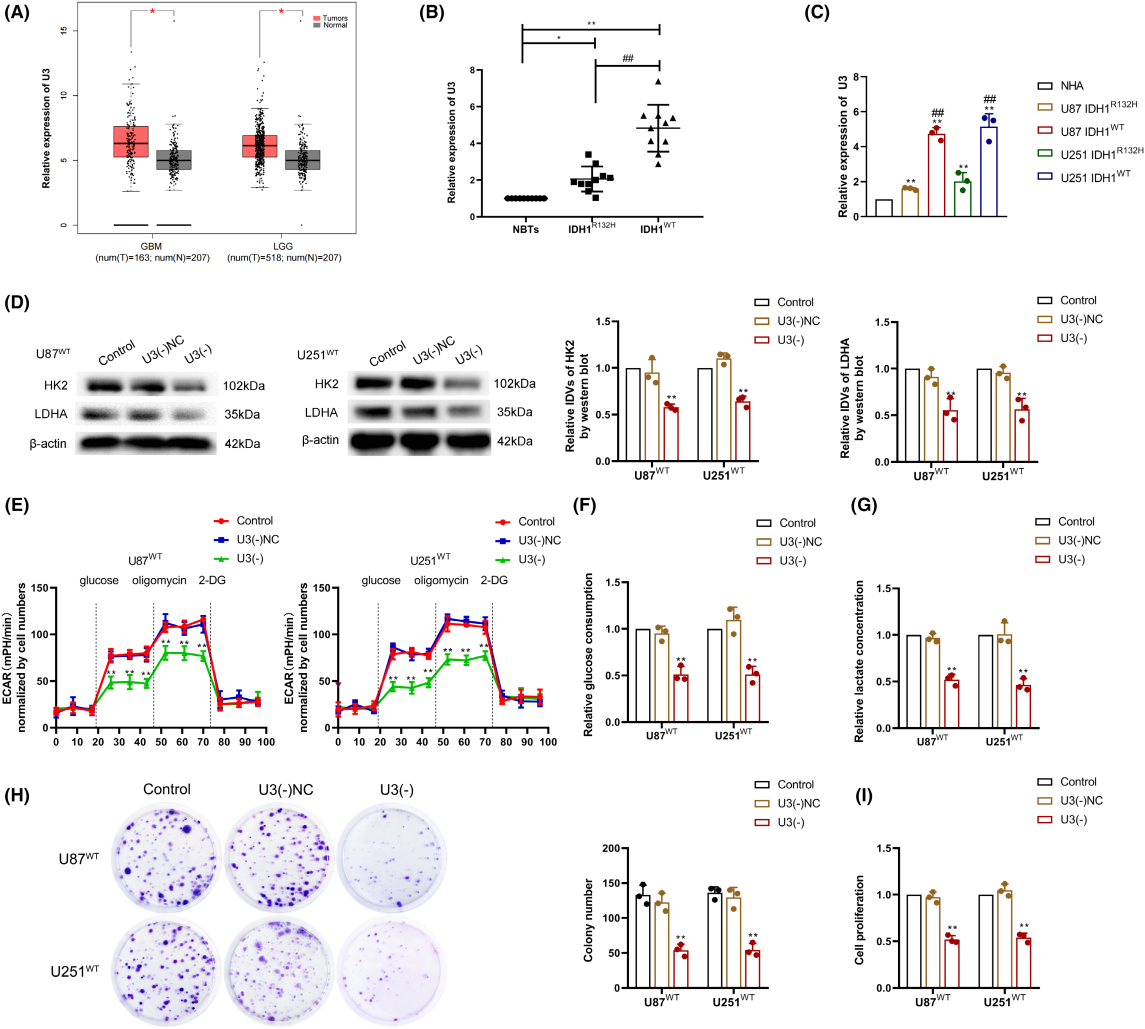
U3 expression was higher in IDH1^WT^ GBM, and the knockdown of U3 suppressed aerobic glycolysis and proliferation. (A) U3 expression in gliomas based on the GEPIA database (B) U3 expression was detected in normal brain tissues (NBTs, *n* = 10), IDH1^R132H^ GBM tissues (*n* = 10), and IDH1^WT^ GBM tissues (*n* = 10) using qRT‐PCR. *p < 0.05 versus NBT group; **p < 0.01 versus NBT group; ^##^ *p* < 0.01 versus IDH1^R132H^ GBM group. (C) U3 expression in normal human astrocytes (NHA), IDH1^R132H^, and IDH1^WT^ GBM cells as determined by qRT‐PCR. **p < 0.01 versus NHA; ^##^ *p* < 0.01 versus IDH1^R132H^ GBM. (D) Hexokinase 2 (HK2) and lactate dehydrogenase A (LDHA) protein expression was detected using western blot after U3 knockdown in IDH1^WT^ GBM cells. (E) Aerobic glycolytic ability was measured after U3 knockdown in IDH1^WT^ GBM cells using an ECAR assay. (F) Glucose consumption was measured after U3 knockdown in IDH1^WT^ GBM cells. (G) Lactate production was measured after U3 knockdown in IDH1^WT^ GBM cells. (H) Proliferation ability was detected after U3 knockdown in IDH1^WT^ GBM cells using a colony formation assay. (I) Cell viability was detected after U3 knockdown in IDH1^WT^ GBM cells using a CCK‐8 assay. **p < 0.01 versus U3(−)NC group. Data are presented as the mean ± SD of three independent experiments per group, unless otherwise specified. Data were statistically analyzed using one‐way ANOVA.

Based on data from Gene Expression Profiling Interactive Analysis (GEPIA, http://gepia.cancer‐pku.cn/index.html), we found that U3 was highly expressed in glioma (Figure 2A). Using qRT‐PCR experiments, we found that U3 expression was significantly elevated in glioma tissues, and U3 expression was higher in IDH1^WT^ glioma tissues when compared to IDH1^R132H^ glioma tissues (Figure 2B). Similarly, qRT‐PCR experiments revealed that U3 expression was significantly higher in IDH1^WT^ GBM cells when compared to normal human astrocyte (NHA) cells, and U3 expression was higher in IDH1^WT^ GBM cells than in IDH1^R132H^ GBM cells (Figure 2C). Based on the above results, we constructed U3 knockdown U87 and U251 IDH1^WT^ GBM cells (Figure S1A) to investigate their effect on the aerobic glycolytic and proliferative capacity of IDH1^WT^ GBM cells. Using western blot assay, we found that HK2 and LDHA expression decreased upon U3 knockdown (Figure 2D), and the extracellular acidification rate (ECAR) assay and colorimetric quantification revealed a significant decrease in the aerobic glycolytic capacity of IDH^WT^ GBM cells upon U3 knockdown (Figure 2E–G). Colony formation and CCK‐8 assays revealed that the proliferative capacity of IDH1^WT^ GBM cells was also reduced upon U3 knockdown (Figure 2H,I). Similarly, we observed that U3 knockdown also inhibited glycolytic capacity and proliferation in IDH1^WT^ GBM cell that overexpressed IDH1^WT^ vector (Figure S2).

### 3.3 U3 overexpression in IDH1^R132H^ GBM cells enhanced glycolytic capacity and proliferation

**FIGURE 3:**
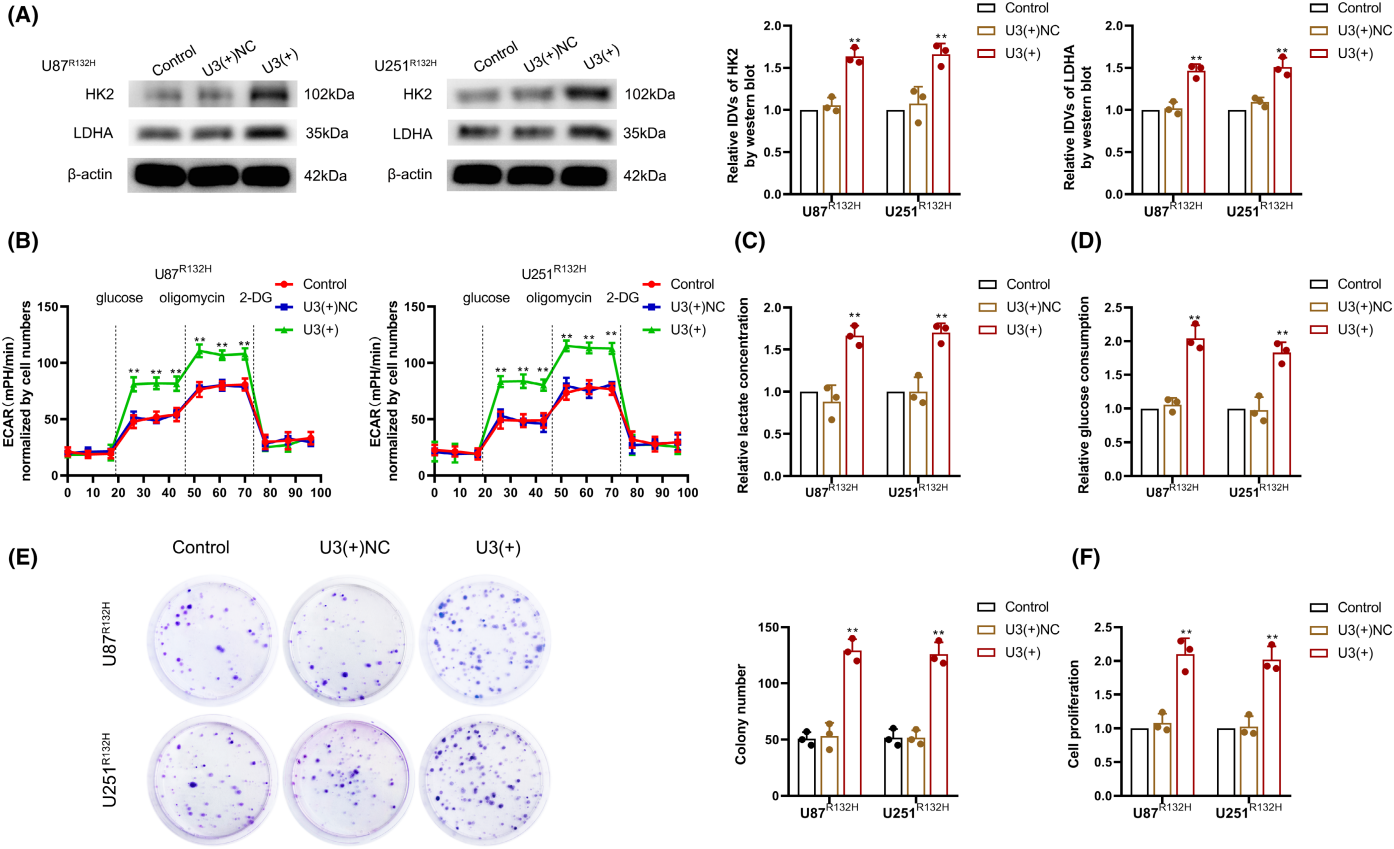
U3 expression was lower in IDH1^R132H^ GBM cells, and the overexpression of U3 enhanced aerobic glycolysis and proliferation. (A) HK2 and LDHA protein expression were detected after U3 overexpression in IDH1^R132H^ GBM cells via western blot. (B) The aerobic glycolytic ability was measured after U3 overexpression in IDH1^R132H^ GBM cells via an ECAR assay. (C) Lactate production was measured after U3 overexpression in IDH1^R132H^ GBM cells. (D) Glucose consumption was measured after U3 overexpression in IDH1^R132H^ GBM cells. (E) Proliferation ability was detected after U3 overexpression in IDH1^R132H^ GBM cells via a colony formation assay. (F) Cell viability was detected after U3 overexpression in IDH1^R132H^ GBM cells via CCK‐8 assay. **p < 0.01 versus U3(+)NC group. Data are presented as the mean ± SD of three independent experiments per group, unless otherwise specified. The data were statistically analyzed via one‐way ANOVA.

In IDH1^R132H^ GBM cells overexpressing U3 (Figure S1B), we detected changes in aerobic glycolysis and proliferative capacity. Western blot analysis revealed significant upregulation of HK2 and LDHA protein expression in IDH1^R132H^ GBM cells upon U3 overexpression (Figure 3A). The ECAR assay and colorimetric quantification revealed that overexpression of U3 significantly promoted aerobic glycolysis, lactate production, and glucose consumption in IDH1^R132H^ GBM cells (Figure 3B–D). The colony formation assay and CCK‐8 assay revealed that U3 overexpression significantly promoted proliferation in IDH1^R132H^ GBM cells (Figure 3E,F).

### 3.4 Hypomethylation of the CpG island in the promoter region of U3 promoted U3 expression in IDH1^WT^ GBM

**FIGURE 4:**
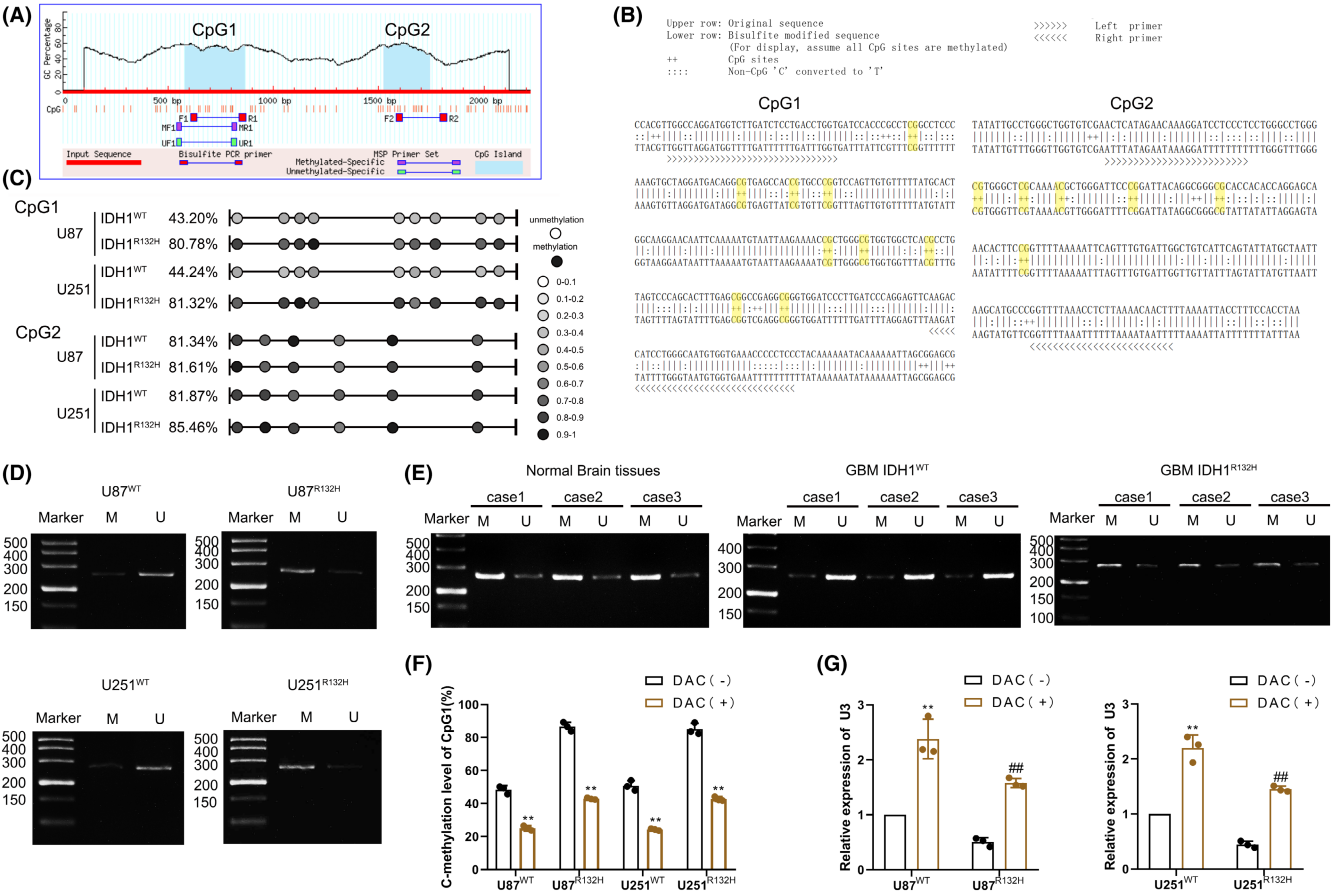
Promoter hypermethylation inhibits U3 expression in IDH1^WT^ GBM. (A) U3 promoter CpG islands were analyzed using the MethPrimer program. (B) Schematic representation of CpG1 and CpG2 bisulfite sequencing and primers. (C) Methylation status of CpG1 and CpG2 at the U3 promoter was detected in IDH1^WT^ and IDH1^R132H^ GBM cells via bisulfite genomic sequencing. (D) Methylation status of CpG1 at the U3 promoter was detected via methylation‐specific PCR in IDH1^WT^ and IDH1^R132H^ GBM cells (M, methylated; U, unmethylated). (E) Methylation status of CpG1 at the U3 promoter was detected via methylation‐specific PCR in normal brain tissues, IDH1^WT^ GBM tissues, and IDH1^R132H^ GBM tissues (M, methylated; U, unmethylated). (F) Change in methylation level of CpG1 in IDH1^WT^ and IDH1^R132H^ GBM cells after treatment with 5‐aza‐2′‐deoxycytidine (DAC). **p < 0.01 versus DAC(−) group. (G) U3 expression was detected in IDH1^WT^ and IDH1^R132H^ GBM cells after treatment with DAC. **p < 0.01 versus IDH1^WT^ DAC(−) group, ^##^ *p* < 0.01 versus IDH1^R132H^ DAC(−) group. Data are presented as the mean ± SD of three independent experiments per group, unless otherwise specified. The data were statistically analyzed via the Student's *t*‐test.

Next, we analyzed the mechanisms that led to the differential expression of U3 in IDH1^WT^ and IDH1^R132H^ GBM cells. The CpG island analysis website (http://www.urogene.org/methprimer/) was used to identify the presence of two CpG islands in the U3 promoter region (Figure 4A). We designed specific primers for pyrosequencing (Figure 4B) to identify whether there was a difference in CpG1 and CpG2 methylation levels in IDH1^WT^ and IDH1^R132H^ GBM cells. We found that the CpG2 methylation status was not significantly different between IDH1^WT^ and IDH1^R132H^ GBM cells; however, CpG1, which is closer to the transcription start site (TSS), was hypomethylated in IDH1^WT^ GBM cells and hypermethylated in IDH1^R132H^ GBM cells (Figure 4C). The methylation‐specific PCR (MSP) assay also indicated that CpG1 methylation in IDH1^WT^ GBM cells was lower than that in IDH1^R132H^ GBM cells (Figure 4D). At the tissue level, we found that CpG1 methylation in IDH1^WT^ glioma tissues was lower than that in normal brain tissues (NBTs), and the level of CpG1 methylation in IDH1^WT^ GBM tissues was lower than that in IDH1^R132H^ GBM tissues (Figure 4E). Upon treatment with 5‐aza‐2′‐deoxycytidine (DAC), CpG1 methylation decreased in both IDH1^WT^ and IDH1^R132H^ GBM cells and U3 expression significantly increased (Figure 4F,G).

### 3.5 U3 downregulated ZBTB7A expression via the formation of sdRNA U3‐miR and enhanced glycolytic capacity and proliferation in IDH1^WT^ GBM cells

**FIGURE 5:**
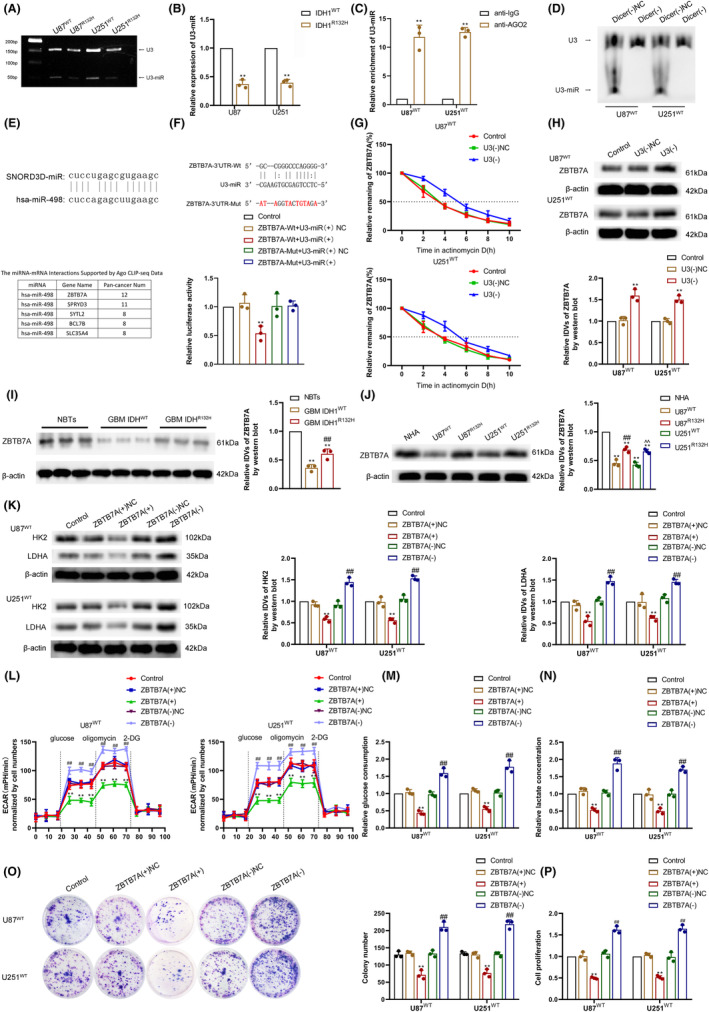
ZBTB7A was downregulated in IDH1^WT^ GBM via U3‐miR targeting of ZBTB7A 3′UTR, and overexpression of ZBTB7A inhibited IDH1^WT^ GBM cell aerobic glycolysis and proliferation. (A) The existence of U3‐miR was detected in IDH1^WT^ and IDH1^R132H^ GBM cells via RT‐PCR. (B) The expression of U3‐miR was analyzed in IDH1^WT^ and IDH1^R132H^ GBM cells via qRT‐PCR. **p < 0.01 versus IDH1^WT^ group. The data were statistically analyzed via the Student's *t*‐test. (C) U3‐miR binding to Ago2 was verified via an RNA immunoprecipitation (RIP) assay. **p < 0.01 versus anti‐IgG group. The data were statistically analyzed via the Student's *t*‐test. (D) The expression of U3 and U3‐miR was detected after Dicer knockdown via northern blot. (E) Schematic representation of the similarity in the sequence of U3‐miR and hsa‐miR‐498 (above). ZBTB7A was a potential target gene of U3‐miR predicted by StarBase database (below). (F) Relative luciferase activity was measured in HEK293T cells co‐transfected ZBTB7A 3′UTR‐Wt or Mut and U3‐miR. **p < 0.01 versus ZBTB7A‐Wt + U3‐miR(+)NC group. (G) ZBTB7A mRNA stability was measured after U3 knockdown in IDH1^WT^ GBM cells treated with actinomycin D. **p < 0.01 versus U3(−) NC group. (H) ZBTB7A protein expression was detected after U3 knockdown via western blot. **p < 0.01 versus U3(−) NC group. (I) ZBTB7A protein expression was detected in NBTs, IDH1^WT^ GBM tissues, and IDH1^R132H^ GBM tissues via western blot. **p < 0.01 versus NBT group; ^##^ *p* < 0.01 versus IDH1^WT^ GBM group. (J) ZBTB7A protein expression was detected in NHA, IDH1^WT^, and IDH1^R132H^ GBM cells via western blot. **p < 0.01 versus NHA group; ^##^ *p* < 0.01 versus U87 IDH1^WT^group; ^^^^ *p* < 0.01 versus U251 IDH1^WT^ group. (K) HK2 and LDHA protein expression were analyzed via western blot in ZBTB7A overexpression or knockdown IDH1^WT^ GBM cells. (L) Aerobic glycolysis was measured via ECAR assay in ZBTB7A overexpression or knockdown IDH1^WT^ GBM cells. (M) Glucose consumption was measured in ZBTB7A overexpression or knockdown IDH1^WT^ GBM cells. (N) Lactate production was measured in ZBTB7A overexpression or knockdown IDH1^WT^ GBM cells. (O) Proliferation ability was detected in ZBTB7A overexpression or knockdown IDH1^WT^ GBM cells via colony formation assay. (P) Viability was detected in ZBTB7A overexpression or knockdown IDH1^WT^ GBM cells via CCK‐8 assay. **p < 0.01 versus ZBTB7A(+)NC group; ^##^ *p* < 0.01 versus ZBTB7A(−)NC group. Data are presented as the mean ± SD of three independent experiments per group, unless otherwise specified. The data were statistically analyzed via one‐way ANOVA.

Several studies have revealed that snoRNA can form snoRNA‐derived RNA (sdRNA) via its stem‐loop structure and is further processed by the Dicer enzyme and bound to the Ago2 protein to exert microRNA‐like functions.^26^, ^27^ RT‐PCR experiments revealed that U3 was able to form the short fragment, sdRNA U3‐miR (Figure 5A), and U3‐miR had an increased expression in IDH1^WT^ GBM cells when compared to IDH1^R132H^ GBM cells (Figure 5B). RIP experiments confirmed that U3‐miR was able to bind to the Ago2 protein (Figure 5C). Knockdown of Dicer in IDH1^WT^ GBM cells (Figure S1C,D) and northern blot experiments revealed no significant change in U3 expression, whereas U3‐miR expression was significantly reduced (Figure 5D). Comparative sequence analysis revealed that U3‐miR was highly similar to hsa‐miR‐496, and by searching and analyzing the starBase database (http://starbase.sysu.edu.cn/), we found that ZBTB7A was the primary target gene (Figure 5E). We further investigated whether U3‐miR has a microRNA‐like function in regulating ZBTB7A expression. We found that U3‐miR possessed a sequence complementary to the 3′UTR of ZBTB7A, and the dual‐luciferase assay demonstrated that U3‐miR could bind to the 3′UTR of ZBTB7A (Figure 5F). The actinomycin D assay revealed that ZBTB7A mRNA stability increased and ZBTB7A protein expression was elevated upon U3knockdown (Figure 5G,H). The above results suggest that U3 promotes the degradation of ZBTB7A mRNA and downregulates ZBTB7A protein expression by forming the short fragment sdRNA U3‐miR to perform microRNA‐like functions. Western blot experiments revealed that ZBTB7A expression was lower in GBM tissues compared with NBTs and ZBTB7A protein expression was significantly lower in IDH1^WT^ GBM than in IDH1^R132H^ GBM (Figure 5I). Furthermore, ZBTB7A protein expression was lower in GBM cells, and ZBTB7A was expressed at significantly lower levels in IDH1^WT^ GBM cells than in IDH1^R132H^ GBM cells (Figure 5J). We further knocked down or overexpressed ZBTB7A in IDH1^WT^ GBM cells (Figure S1E,F) to investigate its effect on the aerobic glycolytic and proliferative capacity of IDH1^WT^ GBM cells. Western blot assay revealed that the knockdown of ZBTB7A in IDH1^WT^ GBM cells significantly promoted the expression of HK2 and LDHA proteins and enhanced cellular aerobic glycolytic capacity and promoted cellular lactate production and glucose consumption, while ZBTB7A overexpression significantly inhibited the expression of HK2 and LDHA proteins and decreased cellular aerobic glycolytic capacity and reduced lactate production and glucose consumption (Figure 5K–N); The proliferative capacity of IDH1^WT^ GBM cells was significantly reduced upon overexpression of ZBTB7A but increased after knockdown of ZBTB7A (Figure 5O,P).

### 3.6 U3 enhanced glycolytic capacity and proliferation of IDH1^WT^ GBM cells via the regulation of ZBTB7A expression

**FIGURE 6:**
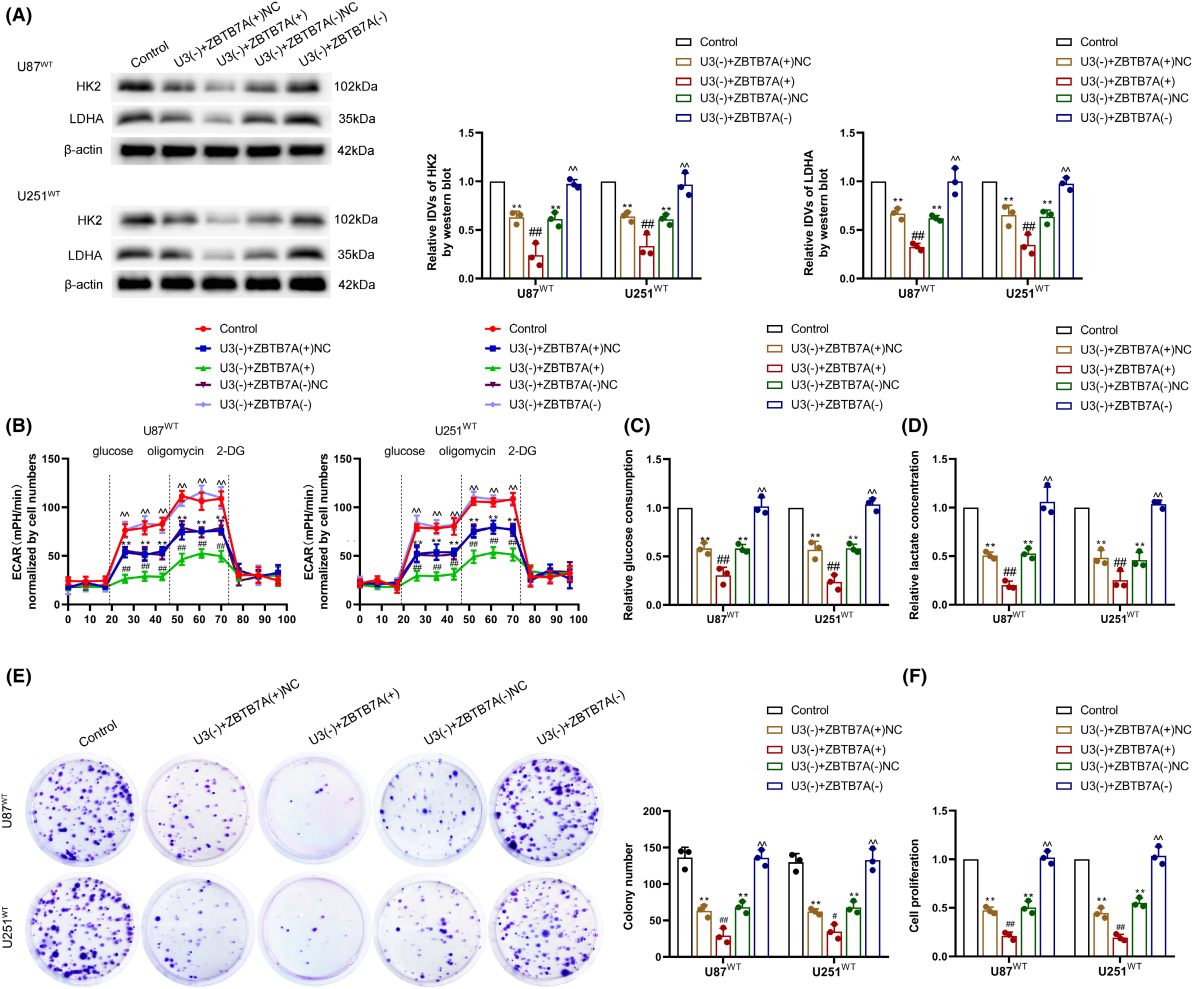
U3 regulated aerobic glycolysis and proliferation in IDH1^WT^ GBM cells via ZBTB7A. (A) Regulation of HK2 and LDHA protein expression by U3 and ZBTB7A was detected via western blot in IDH1^WT^ GBM cells. (B) Regulation of aerobic glycolysis by U3 and ZBTB7A was measured via ECAR assay in IDH1^WT^ GBM cells. (C) Regulation of glucose consumption by U3 and ZBTB7A was measured in IDH1^WT^ GBM cells. (D) Regulation of lactate production by U3 and ZBTB7A was measured in IDH1^WT^ GBM cells. (E) Regulation of proliferation ability by U3 and ZBTB7A was detected via colony formation assay in IDH1^WT^ GBM cells. (F) Regulation of viability by U3 and ZBTB7A was detected via CCK‐8 assay in IDH1^WT^ GBM cells. **p < 0.01 versus control group; ^#^ *p* < 0.05 versus U3(−) + ZBTB7A(+)NC group; ^##^ *p* < 0.01 versus U3(−) + ZBTB7A(+)NC group; ^^^^ *p* < 0.01 versus U3(−) + ZBTB7A(−)NC group. Data are presented as the mean ± SD of three independent experiments per group, unless otherwise specified. The data were statistically analyzed via one‐way ANOVA.

On the basis of the knockdown of U3 expression, we interfered with the expression of ZBTB7A and observed cell glycolytic and proliferation capacity. We detected U3 and ZBTB7A expression via qRT‐PCR or western blot (Figure S3). Compared with the U3 knockdown alone group, overexpression of ZBTB7A after U3 knockdown significantly inhibited the expression of HK2 and LDHA proteins (Figure 6A) and significantly inhibited the aerobic glycolytic capacity of IDH1^WT^ GBM cells (Figure 6B–D). Using colony formation and CCK‐8 assays, we found that overexpression of ZBTB7A after U3 knockdown significantly inhibited the proliferative capacity of IDH1^WT^ GBM cells compared with the U3 knockdown alone group (Figure 6E,F). By contrast, the knockdown of ZBTB7A after the knockdown of U3 had no significant effect on HK2 and LDHA protein expression levels or on the aerobic glycolytic capacity and proliferative capacity of the cells (Figure 6A–F).

### 3.7 ZBTB7A transcriptionally repressed HK2 and LDHA expression by directly binding to the HK2 and LDHA promoter regions

**FIGURE 7:**
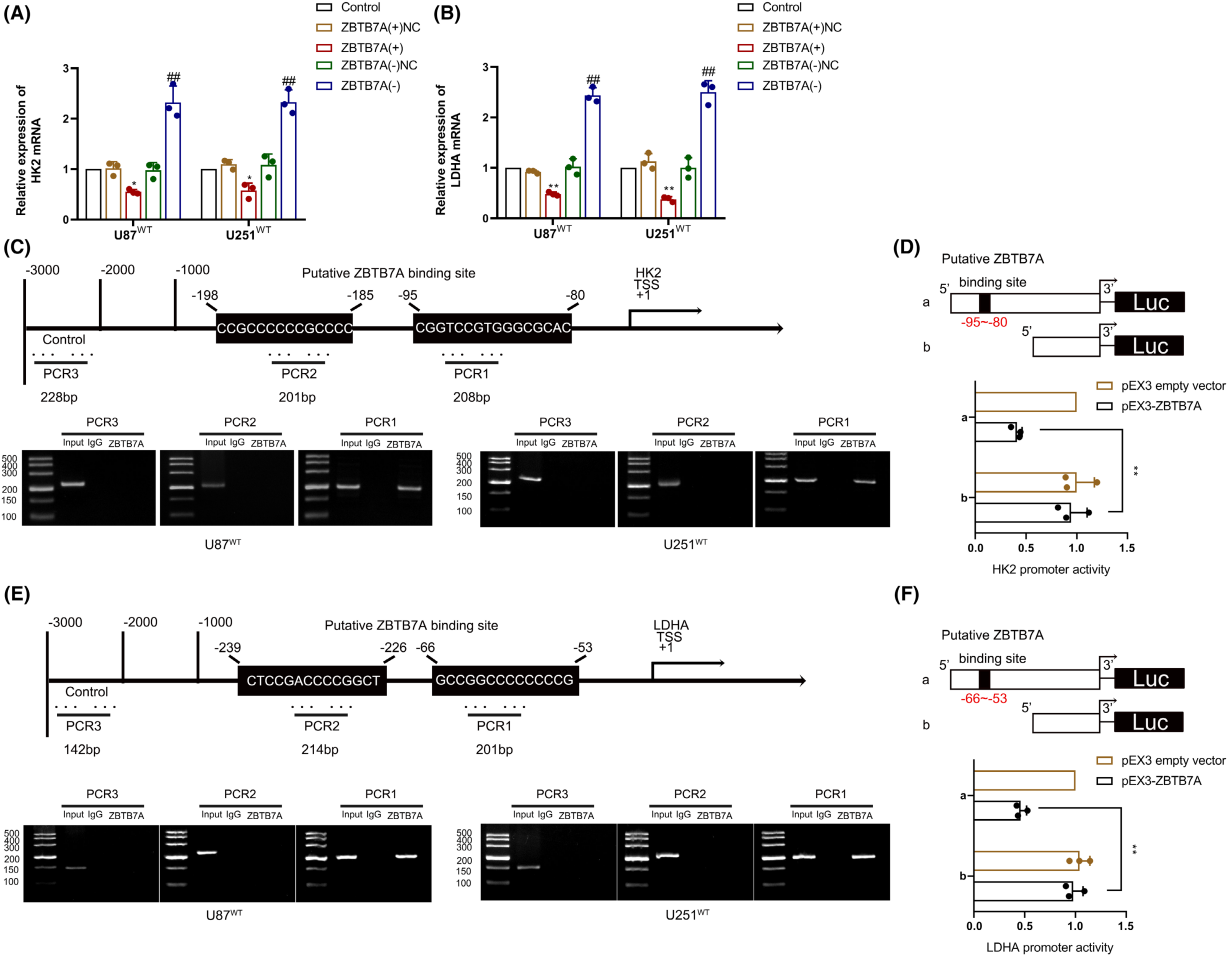
ZBTB7A transcriptionally regulated HK2 and LDHA expression. (A) HK2 mRNA expression was analyzed after ZBTB7A overexpression or knockdown via qRT‐PCR in IDH1^WT^ GBM cells. (B) LDHA mRNA expression was analyzed after ZBTB7A overexpression or knockdown via qRT‐PCR in IDH1^WT^ GBM cells. **p < 0.01 versus ZBTB7A(+)NC group; ^##^ *p* < 0.01 versus ZBTB7A(−)NC group. (C) Diagram showing ZBTB7A binding site of HK2 promoter (above). Chromatin immunoprecipitation (ChIP) assay revealed ZBTB7A bound to the HK2 promoter (below). (D) Schematic diagram of luciferase reporter construction and relative luciferase activity analyzed in cells co‐transfected with pEX3‐ZBTB7A or empty vector and the HK2 promoter (−1000 to 0 bp) (or HK2 promoter without the putative ZBTB7A binding site). (E) Diagram showing ZBTB7A binding site of LDHA promoter (above). ChIP assay revealed ZBTB7A bound to the LDHA promoter (below). (F) Schematic diagram of luciferase reporter construction and relative luciferase activity analyzed in cells co‐transfected with pEX3‐ZBTB7A or empty vector and the LDHA promoter (−1000 to 0 bp) (or LDHA promoter without the putative ZBTB7A binding site). **p < 0.01 versus pEX3 empty vector group. Data are presented as the mean ± SD of three independent experiments per group, unless otherwise specified. The data were statistically analyzed via one‐way ANOVA.

qRT‐PCR experiments revealed that overexpression of ZBTB7A in IDH1^WT^ GBM cells significantly downregulated HK2 and LDHA mRNA expression, whereas the knockdown of ZBTB7A significantly upregulated HK2 and LDHA mRNA expression (Figure 7A,B). Predictions using the Human Transcript Factor Database (http://bioinfo.life.hust.edu.cn/HumanTFDB#!/) revealed potential binding sites for ZBTB7A within the 2000 bp promoter region upstream of the HK2 and LDHA TSS. ChIP experiments using the 3000 bp region upstream of the TSS as a negative control revealed that the negative control did not bind to ZBTB7A, whereas ZBTB7A had a direct binding interaction with HK2 and LDHA in the predicted promoter region binding sites (Figure 7C,E). The luciferase reporter gene assay further demonstrated that ZBTB7A could repress HK2 and LDHA transcriptional expression by binding to their promoter regions (Figure 7D,F).

### 3.8 Knockdown of U3 inhibited the growth of subcutaneous xenograft IDH^WT^ GBM tumor and prolonged the survival of nude mice

**FIGURE 8:**
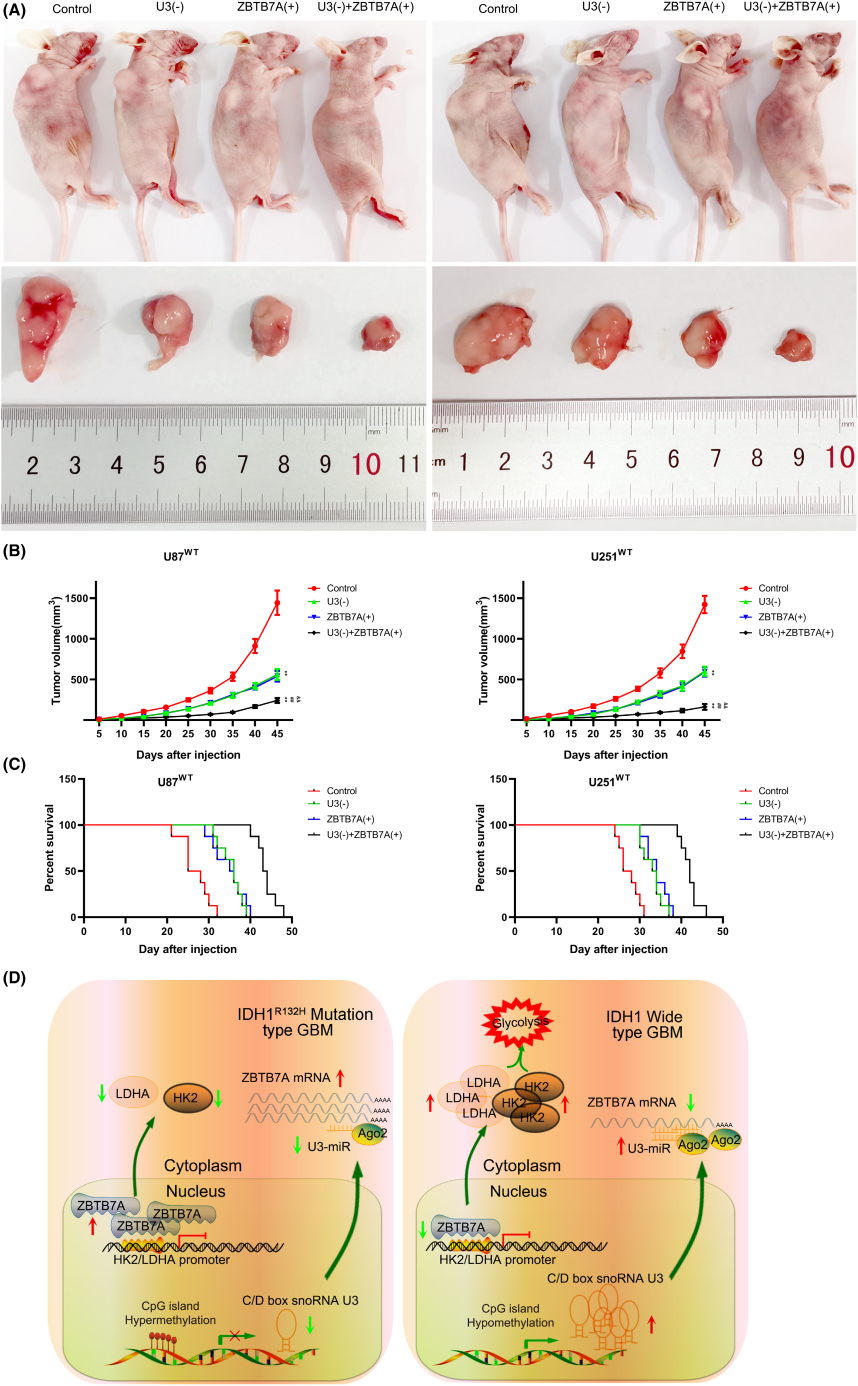
U3 knockdown combined with ZBTB7A overexpression suppressed tumor growth and prolonged survival time in nude mice xenografts. (A) Subcutaneously xenografted nude mice treated with different transfected cells (above). Representative tumors from different groups are shown (below). (B) Tumor growth curve. **p < 0.01 versus control group; ^##^ *p* < 0.01 versus U3(−) group; ^&&^ *p* < 0.01 versus ZBTB7A(+) group. Data are presented as the mean ± SD of eight mice per group and analyzed by two‐way ANOVA. (C) Survival curves of different nude mice groups with orthotopic xenografts. (D) The mechanism of U3/ZBTB7A/HK2 LDHA pathway promoting aerobic glycolysis and proliferation of IDH1^WT^ GBM.

To further demonstrate the effect of U3 knockdown on the inhibition of IDH1^WT^ GBM progression, nude mice were randomly divided into four groups: control, U3(−), ZBTB7A(+), and U3(−) + ZBTB7A(+). The subcutaneous xenograft tumor assay revealed that compared with the control group, the graft tumor volume decreased in the U3(−) and ZBTB7A(+) groups; however, the graft tumor volume was the smallest in the U3(−) + ZBTB7A(+) group (Figure 8A,B). In addition, we injected IDH1^WT^ GBM cells into the right striatal area of nude mice and detected differences in the survival periods of these groups. Compared with the control group, the survival period of nude mice in the U3(−) and ZBTB7A(+) groups was significantly longer and the survival period in the U3(−) + ZBTB7A(+) group was the longest (Figure 8C). Taken together, our results demonstrate the mechanism by which the U3/ZBTB7A/HK2 LDHA pathway promotes aerobic glycolysis and proliferation of IDH1^WT^ GBM cells (Figure 8D).

## 4 DISCUSSION

In this study, we found that the aerobic glycolytic and proliferative capacity of IDH1^WT^ GBM cells were higher than those of IDH1^R132H^ GBM cells. The expression of U3 was higher in IDH1^WT^ GBM cells, and the knockdown of U3 suppressed the glycolytic and proliferative capacity of IDH1^WT^ GBM cells. Furthermore, ZBTB7A expression was lower in IDH1^WT^ GBM cells, and overexpression of ZBTB7A reduced glycolysis and proliferation in IDH1^WT^ GBM cells. U3 downregulated ZBTB7A expression by producing a small fragment of snoRNA, U3‐miR, that bound to the ZBTB7A 3′UTR region and reduced ZBTB7A mRNA stability. Furthermore, ZBTB7A bound directly to HK2 and LDHA promoter regions, thus inhibiting HK2 and LDHA mRNA transcription, downregulating HK2 and LDHA protein expression, and suppressing glycolysis and proliferation of IDH1^WT^ GBM cells. Our study demonstrates the important role of the U3/ZBTB7A/HK2 LDHA pathway in regulating aerobic glycolysis and the proliferative capacity of IDH1^WT^ GBM.

It has been reported that IDH1^WT^ and IDH1^Mut^ molecular phenotypes are closely correlated with glioma tumorigenesis and patient prognosis. Patients with secondary GBM carrying IDH1^R132H^ generally have a better prognosis and longer overall survival than patients with IDH1^WT^ primary glioblastoma (31 months vs. 15 months). Studies have found that the IDH1 molecular phenotype is also closely related to glycolytic energy metabolism in glioma cells, and the aerobic glycolytic capacity of IDH1^WT^ GBM cells is significantly higher than that of IDH1^R132H^ GBM cells,^28^, ^29^ which is consistent with results obtained in this study. The differential expression of the LDHA protein, a key glycolytic enzyme, in different molecular phenotypes of IDH1 may lead to heterogeneity in glycolytic energy metabolism in glioma cells and may affect glioma cell migration, invasion, and proliferation.^25^, ^30^, ^31^ We found that HK2 and LDHA expression was higher in IDH1^WT^ GBM cells than in IDH1^R132H^ GBM cells, and the glycolytic and proliferative capacity of IDH1^WT^ GBM cells were higher than those of IDH1^R132H^ GBM cells.

U3 belongs to the RNU3 gene family of noncoding snoRNAs that cause genomic instability and increase susceptibility to genetic rearrangements, which play crucial roles in tumorigenesis and tumor progression.^16^ Studies have shown that U3A increases the sensitivity of breast cancer cells to 5‐FU chemotherapy by upregulating UMPS expression.^17^ Furthermore, U3 is highly expressed in osteosarcoma cells and promotes cellular resistance to doxorubicin chemotherapy.^32^ It has also been reported that SNORD3B‐1 is highly expressed in hepatocellular carcinoma and is utilized as a diagnostic molecular marker for early‐stage hepatocellular carcinoma and AFP‐negative hepatocellular carcinoma.^33^ This study revealed higher U3 expression in IDH1^WT^ GBM cells than in IDH1^R132H^ GBM cells. Furthermore, the knockdown of U3 inhibited the aerobic glycolytic and proliferative capacity of IDH1^WT^ GBM cells, suggesting that U3 exerts a pro‐oncogenic role via its involvement in abnormal energy metabolism in IDH1^WT^ GBM cells.

snoRNAs can form short RNA fragments of sdRNA via stem‐loop structures. The sdRNAs formed by snoRNAs were found to be of either of two lengths of 17–19 nts or greater than 26 nts.^34^, ^35^ sdRNAs are processed by the Dicer enzyme and bind to the Ago2 protein to perform microRNA‐like functions.^34^, ^36^, ^37^ Studies have shown that SNORA‐93 regulates pipox expression and promotes breast cancer cell invasion by generating miRNA‐like sdRNA‐93.^38^ Furthermore, in prostate cancer, the high expression of sdRNA‐D19b and sdRNA‐A24 enhances prostate cancer cell proliferation, metastatic ability, and resistance to chemotherapy by regulating the expression of CD44 and CDK12.^39^ In this study, we found that U3 could form sdRNA U3‐miR in GBM cells. Consistent with parental U3 expression, U3‐miR had an increased expression in IDH1^WT^ GBM cells when compared to IDH1^R132H^ GBM cells. We found that U3 sdRNA needs to be processed by the Dicer enzyme to form U3‐miR, and U3‐miR can then bind to the Ago2 protein to exert a microRNA‐like function in downregulating ZBTB7A expression.

We also found that ZBTB7A expression was reduced in GBM and was significantly lower in IDH1^WT^ GBM than in IDH1^R132H^ GBM. Moreover, overexpression of ZBTB7A significantly inhibited the aerobic glycolytic and proliferative capacity of IDH1^WT^ GBM cells, whereas the knockdown of ZBTB7A significantly enhanced the aerobic glycolytic and proliferative capacity of IDH1^WT^ GBM cells. As members of the POK (POZ/BTB and Krüppel) family of proteins, ZBTB7A recruits co‐repressors to exert transcriptional repression of target genes.^18^, ^40^ ZBTB7A recruits SMRT and BCOR39 and transcriptionally represses p21 expression by binding to the p21 gene promoter region.^41^ In addition, ZBTB7A negatively regulates androgen receptor‐induced cell proliferation by recruiting NCoR1 and NCoR2 to the androgen response element of the target gene, thus acting as a transcriptional co‐repressor of androgen.^42^ It has been reported that ZBTB7A also acts as a key transcription factor in tumor cells, is closely related to the transcriptional regulation of key glycolytic enzymes, and transcriptionally represses GLUT‐3, PFKP, and PKM2 expression, affecting tumor progression and patient prognosis.^43^, ^44^ In addition to the key glycolytic enzymes mentioned above, we found that overexpression of ZBTB7A in GBM decreased the expression of HK2 and LDHA mRNAs. The results of the ChIP assay and luciferase reporter gene assay indicated that ZBTB4 could directly bind to the promoter region of HK2 and LDHA and exert transcriptional repression to downregulate HK2 and LDHA expression. The inhibition of the aerobic glycolytic energy metabolic pathway could become a new strategy for the treatment of IDH1^WT^ GBM with low ZBTB7A expression.

Finally, the results of the in vivo tumor transplantation experiments in nude mice revealed that the knockdown of U3 alone or overexpression of ZBTB7A significantly inhibited the growth of IDH1^WT^ GBM tumors and prolonged the survival of nude mice; Compared with the knockdown of U3 or overexpression of ZBTB7A alone, a simultaneous knockdown of U3 combined with the overexpression of ZBTB7A significantly inhibited IDH1^WT^ GBM tumors growth, minimized the size of IDH1^WT^ GBM tumors, and prolonged the survival period. The results of the in vivo experiments suggest that the simultaneous knockdown of U3 and overexpression of ZBTB7A have the potential as a treatment of IDH1^WT^ GBM.

In summary, this study demonstrated that U3 is highly expressed and ZBTB7A is expressed at low levels in IDH1^WT^ GBM. We found that U3 formed the sdRNA U3‐miR, which functioned similarly to microRNA by binding to the ZBTB7A 3′UTR region to downregulate ZBTB7A expression. ZBTB7A transcriptionally repressed the expression of HK2 and LDHA and regulated the aerobic glycolytic and proliferative capacity of IDH1^WT^ GBM cells. We revealed a new mechanism by which the U3/ZBTB7A/HK2 LDHA pathway promotes tumorigenesis in IDH1^WT^ GBM cells, thus providing new targets and strategies for the treatment of IDH1^WT^ GBM.

## AUTHOR CONTRIBUTIONS

XL, YX, WD, and YL involved in the study conception and design. WD, DW, CY, HL, JS, and TE involved in the acquisition of data. WD, XR, and LL involved in the analysis and interpretation of data. WD and PW involved in drafting of the manuscript. XL and YL involved in the critical revision of the manuscript for important intellectual content. TM, PW, and YX involved in administrative, technical, and material support. All authors approved the final manuscript.

## FUNDING INFORMATION

This work is supported by grants from the Natural Science Foundation of China (82173071, 82272846); the Liaoning Science and Technology Plan Project (2020‐BS‐097); the Project of Key Laboratory of Neuro‐oncology in Liaoning Province (112‐2400017005); the 2020 Shenyang Research and Development Special Project in Public Health (0‐205‐4‐013); and the Outstanding Scientific Fund of Shengjing Hospital: 201802.

## CONFLICT OF INTEREST STATEMENT

The authors have no relevant financial or nonfinancial interests to disclose.

## Supporting information


Figure S1

Figure S2

Figure S3

Figure S1‐S3

Table S1‐S5

Supporting information S1

## Data Availability

The data that support the findings of this study are available from the corresponding author upon reasonable request.
