# Supplementary figures and images for: U3 snoRNA‐mediated degradation of ZBTB7A regulates aerobic glycolysis in isocitrate dehydrogenase 1 wild‐type glioblastoma cells
Source: CNS Neurosci Ther. 2023 Apr 17;29(10):2811–25. doi: 10.1111/cns.14218 (PMC10493654; doi:10.1111/cns.14218)

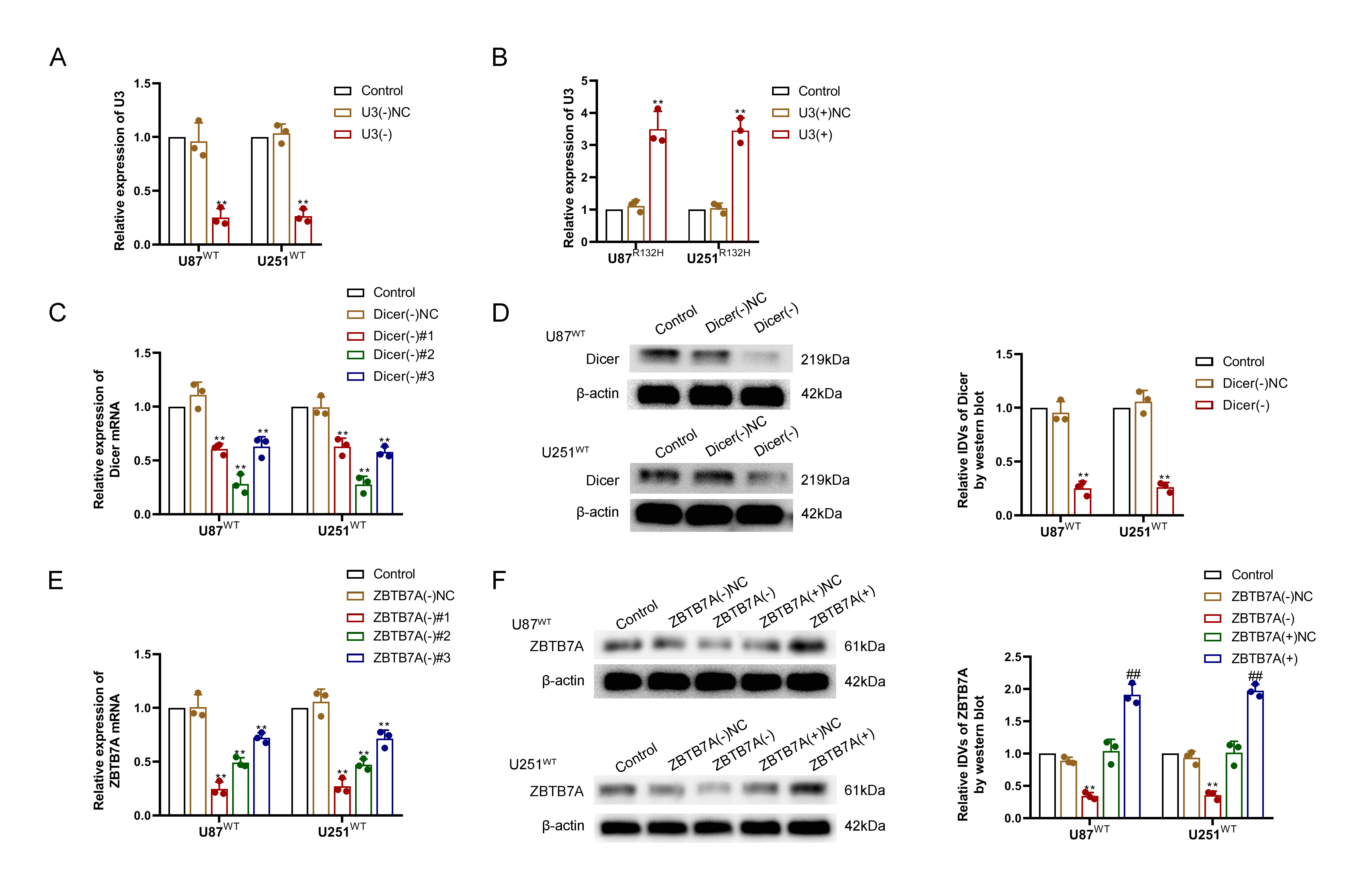

Supplement: Supplementary file 1 — Figure S1 [file CNS-29-2811-s002.tif]

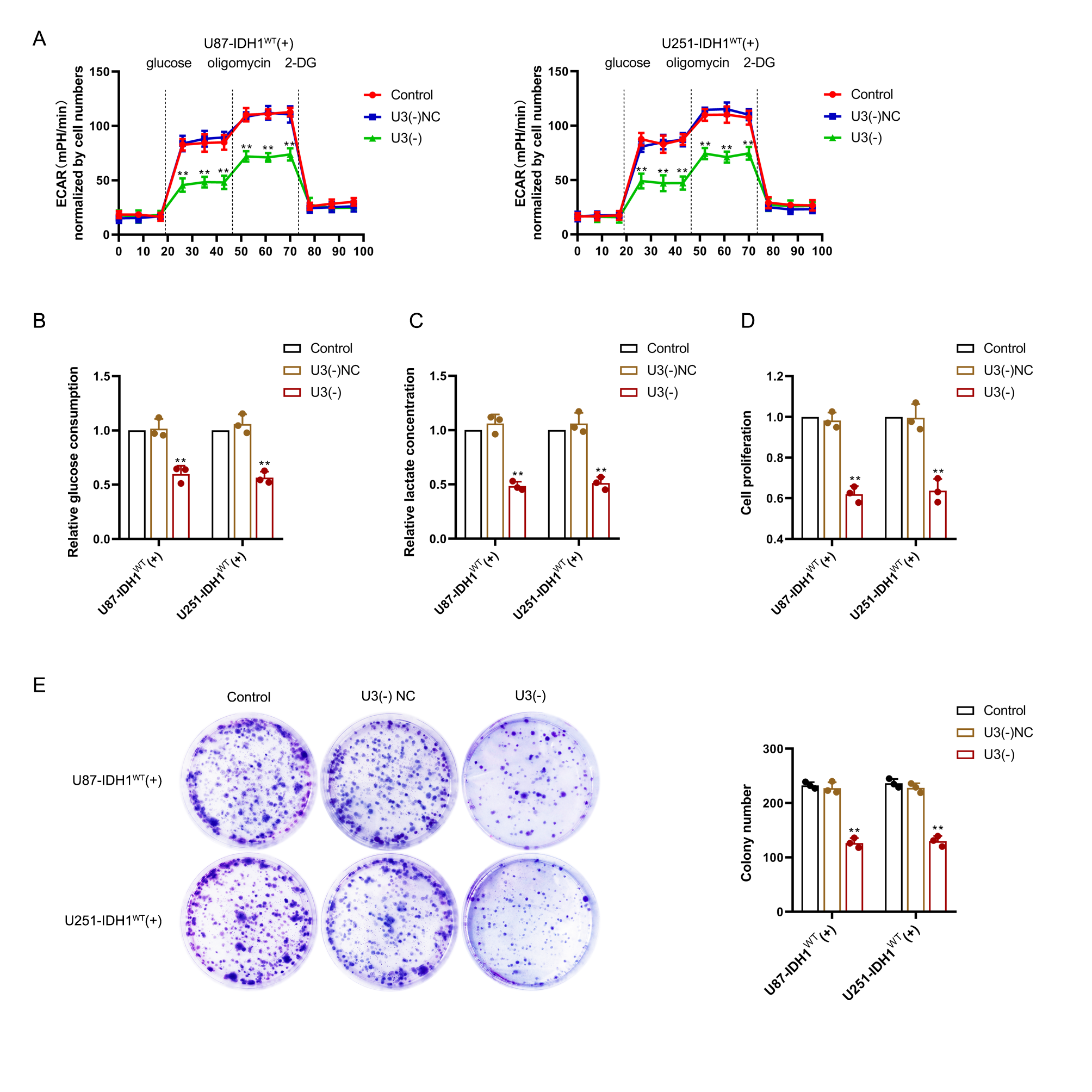

Supplement: Supplementary file 2 — Figure S2 [file CNS-29-2811-s005.tif]

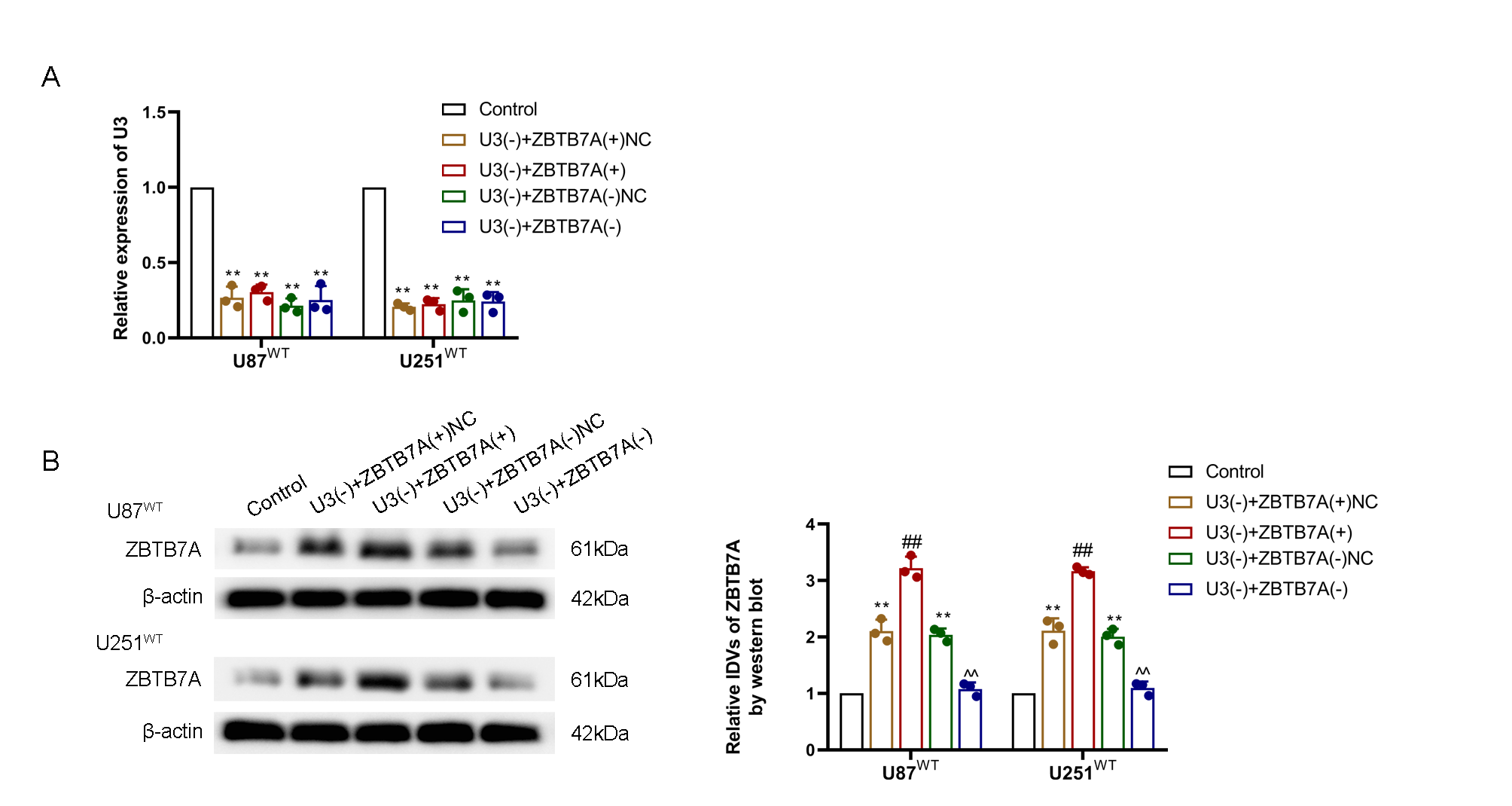

Supplement: Supplementary file 3 — Figure S3 [file CNS-29-2811-s006.tif]
