# Supplementary material for: U3 snoRNA‐mediated degradation of ZBTB7A regulates aerobic glycolysis in isocitrate dehydrogenase 1 wild‐type glioblastoma cells
Source: CNS Neurosci Ther. 2023 Apr 17;29(10):2811–25. doi: 10.1111/cns.14218 (PMC10493654; doi:10.1111/cns.14218)
Supplement: Supplementary file 4 — Figure S1‐S3 [file CNS-29-2811-s003.docx]

**Figure S1**

The results of transfection efficiency.

1. Transfection efficiency of U3 knockdown in IDH1^WT^ GBM cell via qRT-PCR. ^**^*p* < 0.01 versus U3(-)NC group. (B) Transfection efficiency of U3 overexpression in IDH1^R132H^ GBM cell via qRT-PCR. ^**^*p* < 0.01 versus U3(+)NC group. (C) Transfection efficiency of Dicer knockdown (site 1#, site 2#, and site 3#) in IDH1^WT^ GBM cell via qRT-PCR. ^**^*p* < 0.01 versus Dicer(-)NC group. (D) Transfection efficiency of Dicer knockdown (site 2#) in IDH1^WT^ GBM cell via western blot. ^**^*p* < 0.01 versus Dicer(-)NC group. (E) Transfection efficiency of ZBTB7A knockdown (site 1#, site 2#, and site 3#) in IDH1^WT^ GBM cell via qRT-PCR. ^**^*p* < 0.01 versus ZBTB7A(-)NC group. (F) Transfection efficiency of ZBTB7A knockdown (site 1#) and overexpression in IDH1^WT^ GBM cell via western blot. ^**^*p* < 0.01 versus ZBTB7A(-)NC group; ^##^*p* < 0.01 versus ZBTB7A(+)NC group. Data are presented as the mean±SD of three independent experiments per group, unless otherwise specified. The data were statistically analyzed using one-way ANOVA.

**Figure S2**

Knockdown of U3 suppressed aerobic glycolysis and proliferation in IDH1^WT^ overexpression GBM cell

(A) Aerobic glycolytic ability was measured after U3 knockdown in IDH1^WT^(+) GBM cells using ECAR assay. (B) Glucose consumption was measured after U3 knockdown in IDH1^WT^(+) GBM cells. (C) Lactate production was measured after U3 knockdown in IDH1^WT^(+) GBM cells. (D) Cell viability was detected after U3 knockdown in IDH1^WT^(+) GBM cells using CCK-8 assay. (I) Proliferation ability was detected after U3 knockdown in IDH1^WT^(+) GBM cells using colony formation assay. ^**^*p* < 0.01 versus U3(-)NC group. Data are presented as the mean±SD of three independent experiments per group, unless otherwise specified. Data were statistically analyzed using one-way ANOVA.

**Figure S3**

The expression of U3 and ZBTB7A

1. The expression of U3 was detected via qRT-PCR. ^**^*p* < 0.01 versus control group. (B) The expression of ZBTB7A protein was detected via western blot. ^**^*p* < 0.01 versus control group; ^##^*p* < 0.01 versus U3(-)+ZBTB7A(+)NC group; ^^^^*p* < 0.01 versus U3(-)+ZBTB7A(-)NC group. Data are presented as the mean±SD of three independent experiments per group, unless otherwise specified. Data were statistically analyzed using one-way ANOVA.
