# Supplementary material for: U3 snoRNA‐mediated degradation of ZBTB7A regulates aerobic glycolysis in isocitrate dehydrogenase 1 wild‐type glioblastoma cells
Source: CNS Neurosci Ther. 2023 Apr 17;29(10):2811–25. doi: 10.1111/cns.14218 (PMC10493654; doi:10.1111/cns.14218)
Supplement: Supplementary file 5 — Table S1‐S5 [file CNS-29-2811-s001.docx]

**Tables**

Table S1. The primers of target RNA in quantitative real-time PCR (qRT-PCR)

| Gene | Primer | Sequence |
| --- | --- | --- |
| LDHA | Forward | AGGTGATCAAACTCAAAGGCTA |
|  | Reverse | CCCAAAATGCAAGGAACACTAA |
| HK2 | Forward | CGACAGCATCATTGTTAAGGAG |
|  | Reverse | GCAGGAAAGACACATCACATTT |
| U3 | Forward | CTTTCTGGCGTTGCTTGGC |
|  | Reverse | CTCTCCCTCTCACTCCCCAA |
| U3-miR | Forward | TTGGTCGCTCCTGAGCGTGA |
|  | Reverse | ATCCAGTGCAGGGTCCGAGG |
|  | RT Primer | GTCGTATCCAGTGCAGGGTCCGAGGTATTCGCACTGGATACGACGCTTCA |
| Dicer | Forward | GTTTGAAAGCGTTGAGTGGTAT |
|  | Reverse | GCCAGCTTCCTTTATCAATCTG |
| ZBTB7A | Forward | AGCTGGACCTTGTAGATCAAAT |
|  | Reverse | CATCCAGGTCATCATCGGAC |
| IDH1 | Forward | GATGGCAAGACAGTAGAAGCAGAGG |
|  | Reverse | ATGGAAGCAATGGGATTGGTGGAC |
| β-actin | Forward | CCTGGCACCCAGCACAAT |
|  | Reverse | GGGCCGGACTCGTCATAC |

TableS2 The short hairpin RNAs against Dicer and ZBTB7A sites

| gene | Site | Sequence(5’-3’) |
| --- | --- | --- |
| Dicer | #1 | GGAAGAGGCTGACTATGAA |
|  | #2 | GATCCTATGTTCAATCTAAA |
|  | #3 | GAGTGTTTGAGGGATAGTT |
| ZBTB7A | #1 | AGCCAGTACTTCAAGAAGC |
|  | #2 | GACCTTGTAGATCAAATTGA |
|  | #3 | TGGACTACTACCTGAAGTAC |

Table S3. Sequences of sgRNA for knockdown of U3

| sgRNA1 | GTAGAGCACCGAAAACCACG |
| --- | --- |
| sgRNA2 | AGAAAGCCGGCTTCACGCTC |

Table S4. Primer for methylation specific PCR (MSP)

| Primer | Sequence | Product size |
| --- | --- | --- |
| Left M primer | TATAGGCGTAGGTTATTACGTTCGG |  |
| Right M primer | ATCAAAAAATCCACCCGCCTCGAC | 284 |
| Left U primer | TATAGGTGTAGGTTATTATGTTTGG |  |
| Right U primer | AAATCAAAAAATCCACCCACCTCAAC | 286 |

Table S5. Primers used for ChIP experiments

| Gene | Binding site or Control | Forward | Reverse | Product size(bp) |
| --- | --- | --- | --- | --- |
| HK2 | PCR1 | F:GTGAGCGATGATTGGCTGC | R:CGCGGATTTTCTTAGCTGGG | 208 |
|  | PCR2 | F:TTTTTCCAGTCGCCCCACAC | R:GCCGCTCAGACACCGGAG | 201 |
|  | PCR3 | F:CAAATTTTGGGGCCTGCCTG | R:TGCCCTTAGACACCTCCTGA | 228 |
| LDHA | PCR1 | F:GGAGGGCAGCACCTTACTTA | R:GGAGGGGCCTTAAGTGGAAC | 201 |
|  | PCR2 | F:CCTCCCCAGGTTTCATGGAT | R:CACGTGTGAGTCGGGCTG | 214 |
|  | PCR3 | F:GATACACCCATTGGCACCCA | R:GCTGACTAACCCCAAAGCCT | 142 |
