# Supplementary material for: U3 snoRNA‐mediated degradation of ZBTB7A regulates aerobic glycolysis in isocitrate dehydrogenase 1 wild‐type glioblastoma cells
Source: CNS Neurosci Ther. 2023 Apr 17;29(10):2811–25. doi: 10.1111/cns.14218 (PMC10493654; doi:10.1111/cns.14218)
Supplement: Supplementary file 6 — Supporting information S1 [file CNS-29-2811-s004.docx]

**Supporting information materials and methods**

**Clinical specimens**

Normal human brain specimens (NBTs, n=10) and IDH1^WT^ GBM and IDH1^R132H^ GBM tissues were obtained from the Department of Neurosurgery, Shengjing Hospital of China Medical University. Glioma specimens were diagnosed by two experienced pathologists and graded according to the WHO classification of glioma. NBTs were obtained from fresh autopsy materials (donation of individuals who died in traffic accident and free of any prior pathologically detectable condition). All specimens were preserved in liquid nitrogen until use.

**Cell lines**

Human glioma cell lines (U87 and U251) and Human embryonic kidney (HEK) 293 T cells were cultured in Dulbecco’s modified Eagle’s medium (DMEM, HyClone, USA) supplemented with 10% fetal bovine serum (FBS, Gibco, Grand Island, NY, USA). Normal human astrocyte (NHA) cells were cultured in RPMI-1640 medium (Gibco, Grand Island, NY, USA) with 10% FBS. All cells were cultured at 37℃ with 5% CO_2_ in incubator.

**Quantitative real-time PCR (qRT-PCR)**

After total RNA was extracted from cells and tissues with Trizol reagent, we used Nanodrop Spectrophotometer (ND-100, Thermo, USA) to measure the RNA concentration via 260/280nm absorbance. PrimeScript^TM^ RT-PCR Kit (TakaraBio, Inc., Japan) was utilized to detect the expression of target RNAs and Bulge-Loop^TM^ miRNA qRT-PCR system (Ribobio, Guangzhou, China) was utilized to detect the expression of target U3-miR via 7500 Fast real-time PCR system (Applied Biosystems, USA). β-actin was used as internal reference to detect the expression of mRNA, and U6 was used as internal reference to detect the expression of U3 and U3-miR. The expression levels were normalized to the endogenous controls and calculated as fold change using the relative quantification(2^-∆∆^Ct) method.

**Western blot**

RIPA buffer and protease inhibitors (Beyotime Institute of Biotechnology, Shanghai, China) were used to lyse the cells. Cell proteins were extracted by centrifugation and its concentration was analyzed by BCA protein assay (Beyotime Institute of Biotechnology, Shanghai, China). Equal amount of protein samples were separated by electrophoresis on 10% SDS/PAGE gels and then transferred to PVDF membranes. PVDF membranes were blocked in 5% nonfat milk in tris-buffered saline (TBS) containing 0.1% Tween-20 (TTBS) for 2h at room temperature and then were incubated with primary antibodies overnight at 4℃as follows: HK2(1:5000; Proteintech, Chicago, IL, USA), LDHA (1:5000; Proteintech, Chicago, IL, USA), IDH1-R132H(1:200; Dianova, Hamburg, Germany), β-actin(1:5000; Proteintech, Chicago, IL, USA), ZBTB7A(1:10,000, Abcam, Cambridge, MA, USA), Dicer(1:1000; Proteintech, Chicago, IL, USA). Then, the membranes were incubated with the secondary antibody goat anti-mouse (1:10,000; Proteintech, Chicago, IL, USA) or goat anti-rabbit (1:10,000; Proteintech, Chicago, IL, USA). The enhanced chemiluminescence (ECL) kit (Beyotime Institute of Biotechnology, Shanghai, China) and ChemImager 5500 V2.03 software were utilized to visualize the protein bands. The relative integrated density values (IDV) were calculated using Image-J software based on β-actin as endogenous control.

**Cell transfection**

Cells were seeded in 24-well plates and transfected with the indicated plasmids using Lipofectamine 3000 reagent (Life Technologies, Carlsbad, CA, USA) following manufacture’s protocol. Then, the stably transfected cells were selected via incubation with puromcin, blastmycin, or G418 (Sigma-Aldrich, StLouis, MO, USA). The resistant cell clones were harvested 4 weeks after transfection.

**Extracellular acidification rate**

XF glycolysis stress test kit (Seahorse Bioscience, USA) was utilized to measure extracellular acidification rate according to manufacture’s instruction. 5×10^4^ targeted cells per well were seeded into Seahorse plates and 500μL medium was added and cultured for overnight. Next day, the culture solution was discarded. XF Base Medium was added to each well, and the cells were incubated for 2h in starvation condition. Cells were treated with glucose (10nM), oligomycin(1μM) and 2-deoxyglucose (50nM) in the XF24 Extracellular Flux Analyzer (Seahorse Bioscience, Billerica, MA, USA) to measure ECAR.

**Glucose utilization and lactate production assays**

Lactate detection kit and glucose detection kit were purchased from Jiancheng Bioengineering Institution (Jiancheng, Nanjing, China). 2×10^4^ targeted cells per well were seeded in 96-well plate with 200 µl medium. After 48h incubation period, the culture medium was collected and lactate and glucose were measured using colorimetric method according to the manufacture’s protocol.

**CCK-8**

Cell Counting kit-8 (CCK-8) assay kit (Beyotime Institute of Biotechnology, Shanghai, China) was utilized to measured the viability of cell according to manufacture’s protocol. 2×10^3^ cells were seeded in 96-well plates. After the cells adhering to the wall, removed culture medium and added 90µl medium+10µl CCK-8 in each well. After 2h incubation period, the absorbance at the wavelength of 450nm was detected to measure cell proliferation via SpectraMax M5 microplate reader (Molecular Devices, USA).

**RNA immunoprecipitation assay**

The RNA immunoprecipitation assay (RIP) experiment was carried out according to the manufacturer's instructions using an EZ-Magna RNA-binding protein immunoprecipitation kit (Millipore, USA). The RIP assay was performed with an Ago2 antibody (Proteintech, Chicago, IL, USA) and IgG as a negative control. The lysate of the cells was treated with RIP buffer, magnetic beads, and antibody. After that, proteinase K was added to the mixture, and immunoprecipitated RNA was extracted. The concentration of RNA was determined using a Nanodrop Spectrophotometer (ND-100, Thermo, USA). Finally, qRT-PCR was used to confirm the existence of the binding targets RNA.

**Chromatin immunoprecipitation assay**

The chromatin immunoprecipitation (ChIP) experiment was performed according to the manufacturer's protocol using the Simple ChIP Enzymatic Chromatin IP Kit (Cell Signaling Technology, Danvers, MA, USA). Formaldehyde was used to cross-link proteins and DNA in glioma cells for 10 minutes before being terminated with glycine. The chromatin was digested with micrococcal nuclease after the cells were collected in lysis buffer. 2% lysates were utilized as an input reference control and the other lysates were incubated with anti-ZBTB7A antibody or normal IgG antibody with rotation and stored at -20°C. NaCl and proteinase K were used to break down DNA crosslinks, and the immunoprecipitated DNA was then purified. PCR primers were used to amplify anti-ZBTB7A antibody and anti-IgG antibody immunoprecipitated DNA.

**Dual-luciferase reporter assay**

HEK293T cells were seeded into a 96-well plate. ZBTB7A 3’-UTR (ZBTB7A-Wt, ZBTB7A-Mut containing mutation in the predicted binding site of U3-miR) luciferase reporter gene vector was constructed. The indicated vectors were co-transfected with U3-miR and U3-miR NC, respectively. To analyze how ZBTB7A regulated HK2 and LDHA transcriptional expression by binding to their promoter regions, the pmirGLO dual-luciferase vector (Promega, Madison, WI, USA) was employed as a control. The wild type vector containing promoter region sequences of HK2, and LDHA and mutant type vector containing truncated sequences were amplified by PCR respectively. The PCR products were subcloned into the pGL3-Basic vector (Promega, Madison, WI, USA) to obtain different recombinant vectors. pEX3-ZBTB7A recombinant vector (GenePharma, Shanghai, China) containing human full-length ZBTB7A sequence was also constructed. The HEK-293T cells were co-transfected with the recombinant wild type or mutant luciferase expression vector and pEX3-ZBTB7A or empty vector. The luciferase activity was detected at 48h after co-transfection using Dual-Luciferase reporter assay kit (Promega, Madison, WI, USA) following the manufacturer’s protocol.

**Tumor xenograft in nude mouse**

All the animal experiments were performed following the Animal Welfare Act and approved by the Ethics Committee of China Medical University. Four-week-old athymic nude mice (BALB/c) were purchased from the Beijing HFK Bioscience co.,Ltd. (Beijing, China). The nude mice were divided into four groups: Control, U3(-), ZBTB7A(+), and U3(-) +ZBTB7A(+). Mice were randomly divided into each double-blind group by two performers, n = 8 per group. For subcutaliuneous implantation, 3×10^6^ cells were injected subcutaneously under right axilla area and tumor nodules were estimated with caliper at a 5-day interval. Mice were scarified on the 45th day after injection, and the tumors volumes =(length × width^2^)/2. In survival study, 3×10^6^ cells were injected into the right striatum. The number of survival mice was recorded every day and survival analysis was performed according to Kaplan-Meier survival curve.
